# The American Meat Science Association and *Animal Frontiers*: 10 yr of partnership

**DOI:** 10.1093/af/vfaa032

**Published:** 2020-10-30

**Authors:** Anna C Dilger

**Affiliations:** Department of Animal Sciences, University of Illinois, Urbana-Champaign, IL

Food is a significant part of our lives. It provides us the nutrients we need to survive, but also connects us with other people. Many of us have memories that involve food like eating traditional meals with our grandparents, a romantic dinner with our partner, or a cook-out to celebrate summer. For many people, food is how we express our caring and love for other people and explore the world through global cuisine. But because food is so personal, it can also be controversial. Many people are far removed from the processes of animal agriculture and the food industry. This distance, along with many different agendas, has allowed misconceptions and mistrust to grow between the public who consumes food and those involved in its production.

This is why is it imperative that we, the scientists and industry leaders of the American Meat Science Association (AMSA) along with our partner organizations, strive to close that gap through better communication and transparency around controversial and emerging issues. *Animal Frontiers* is one avenue we use to narrow the distance between us and the food-consuming public. It provides a forum where scientists can highlight new research and technologies as well as directly address the emerging issues and controversies of food production. The goal is to connect with the global community and policy-makers. It is vital that we bridge the distance between food producers and consumers. Recent agricultural food shortages have demonstrated how important that linkage is and how few consumers understand the long and complex chain of global meat production.

Over the last 10 yr, AMSA has led the charge in producing articles focused on technologies used in livestock and meat production, advancements in food safety, the role of meat in healthy diets and as a means to combat food insecurity, as well as the clearing defining the term meat (Figure 1). We also appreciate the efforts of the other member societies in highlighting issues along the production chain to help us present a well-rounded source of information to the public. Together, through *Animal Frontiers*, we can reverse the trend of growing mistrust around food and deliver directly to the public timely and accurate scientific information to aid in their decision making.

**Figure 1.**
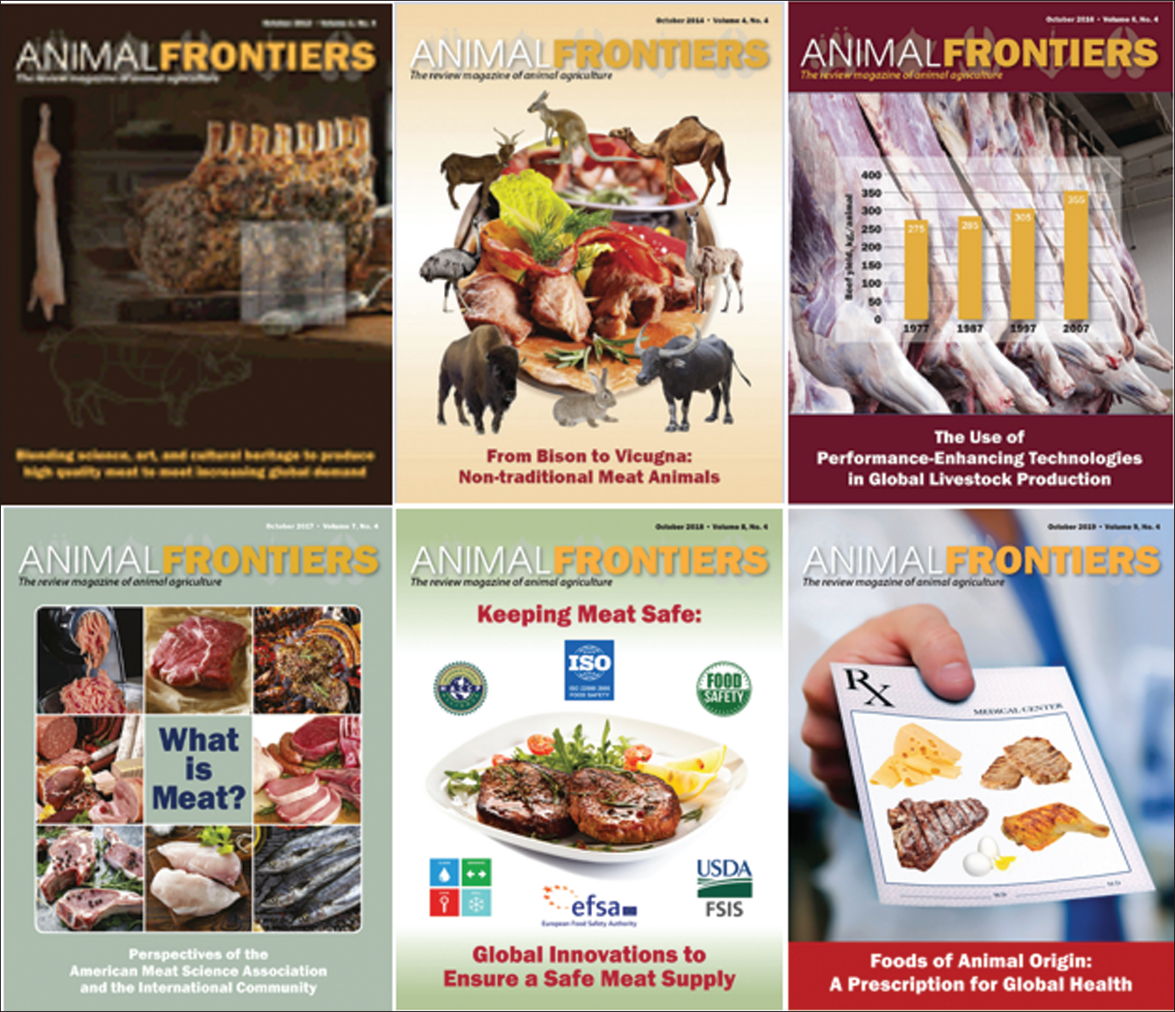
*Animal Frontiers* issues led by the American Meat Science Association (AMSA).

## About the Author

Anna Dilger, Ph.D., earned her B.S. and M.S. at Purdue University and her Ph.D. at the University of Illinois, where she is currently an associate professor in the Department of Animal Sciences. The focus of her research is on muscle biology, especially the mechanisms regarding increased animal growth, efficiency, and their effects on meat quality. Anna has served on the Management Board for *Animal Frontiers* representing the American Meat Science Association since 2017. Prior to that, she served as both a guest editor (2016) and author (2015) for the journal.

